# Asymptomatic gastric tuberculosis in the gastric body mimicking an isolated microscopic erosion: A rare case report

**DOI:** 10.1097/MD.0000000000028888

**Published:** 2022-02-25

**Authors:** Wenguang Zhang, Fusheng Song, Zhimei Zhang, Jun Yang, Linlin Zhao

**Affiliations:** a Department of Gastroenterology, Banan Hospital of Chongqing Medical University (People's Hospital of chongqing Banan District), Chongqing, China; b Department of General Practice, Banan Hospital of Chongqing Medical University (People's Hospital of chongqing Banan District), Chongqing, China.

**Keywords:** case report, gastric erosion, gastric tuberculosis, gastroscopy

## Abstract

**Introduction:**

Gastric tuberculosis is rarely seen in clinical practice, which occurs mostly secondary to lung tuberculosis, intestinal tuberculosis, and other common tuberculosis. Gastric tuberculosis rarely presents as a single microscopic superficial erosion. We recently diagnosed such a case, hence reporting it herein.

**Patient concerns:**

A 40-year-old female patient was admitted with a chief complaint of painful enlarged cervical lymph nodes. She had no other symptoms or any previous history of remarkable diseases.

**Diagnosis:**

Physical examination found multiple enlarged cervical lymph nodes. Computer tomography revealed multiple circular well-defined soft tissue masses in the bilateral carotid sheath spaces. A cervical lymph node biopsy showed caseous necrosis with infiltration of neutrophils and lymphocytes, and most importantly, mycobacteria through staining for acid fast bacilli. Routine gastroscopy showed a 0.5 cm × 0.5 cm well-defined erosion on the large curvature of the gastric body. Gastric biopsy revealed chronic granulomatous inflammation with mycobacteria through staining for acid fast bacilli. The patient was diagnosed as having cervical lymph node tuberculosis and gastric tuberculosis.

**Interventions and outcomes:**

She received 6 months of standard anti-tuberculosis therapy. The enlarged cervical lymph nodes shrank in size and the pain was relieved.

**Conclusions:**

Gastroscopy should be performed to look for gastric tuberculosis if the patient presents primary tuberculosis in other organs/tissues such as cervical lymph nodes. If any small erosion is found, a biopsy is justified for checking the possibility of gastric tuberculosis.

## 1 Introduction

Gastric tuberculosis is rarely seen in clinical practice, which occurs mostly secondary to lung tuberculosis, intestinal tuberculosis, and other common tuberculosis. Endoscopic manifestations of gastric tuberculosis are not specific.^[1,2]^ Gastric tuberculosis often presents as an irregular erosion, ulcer, submucosal bulge lesions, or gastric sinus deformation.^[3–6]^ It is rare to present as a single microscopic superficial erosion. Here, we report such a case of gastric tuberculosis in a patient with cervical lymph node tuberculosis.

## 2 Case presentation

**Figure 1:**
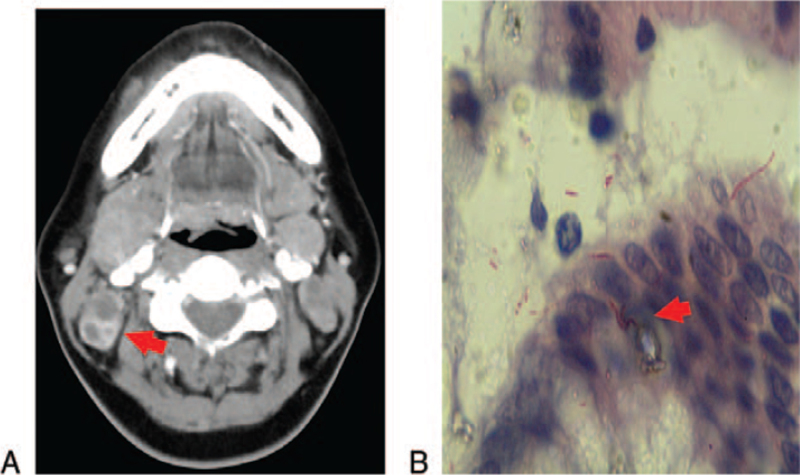
CT images of the patient's neck and acid-fast staining results of cervical lymph node biopsy. A. Enhancement CT image showed the uneven intensity of the lesions and the limited low-density shadow (shown by arrow) in the neck. B. Right cervical lymph node biopsy stained positive for acid-fast bacilli (magnification, 1000×). CT = computed tomography.

**Figure 2:**
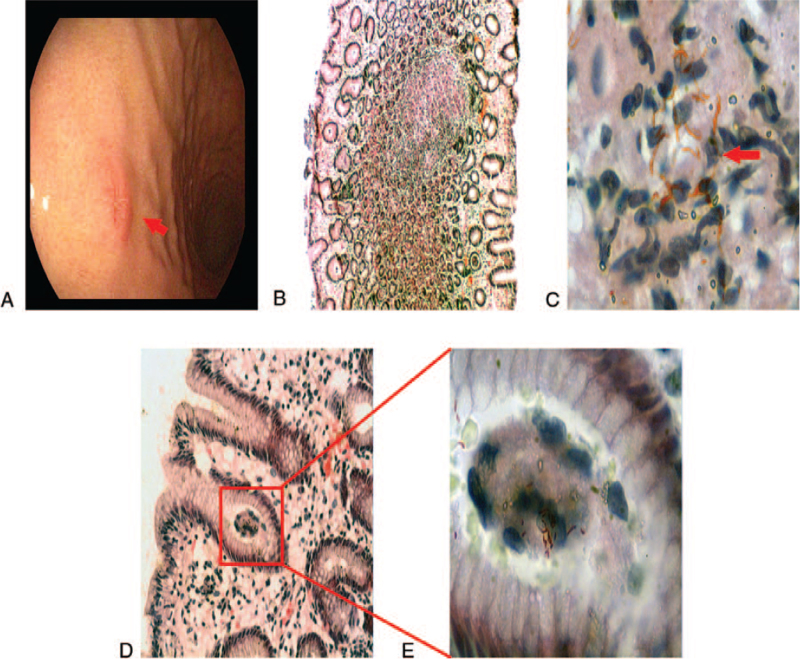
Gastroscopy and pathological manifestations. A. Gastroscopy (white light) shows about 0.5 cm × 0.5 cm superficial elevation of the upper gastric bend. B and D. Gastric biopsy with hematoxylin and eosin staining shows mild chronic gastric mucosa erosion with edema and focal granulomatitis (magnification, 40×). C and E. Gastric biopsy stained positive for acid-fast bacilli (magnification, 1000×).

A 40-year-old female patient was admitted to our hospital on April 23, 2021. Her chief complaint was painful enlarged cervical lymph nodes for 2 weeks. She had no other symptoms or any previous history of remarkable diseases. Physical examination found multiple enlarged cervical lymph nodes, the largest of which had a size of approximately 3 cm × 2.5 cm, with well-defined boundaries. The enlarged lymph nodes were fixed, solid on palpation, and without any ulcer over the skin. Routine laboratory tests did not show any abnormalities except for an increased erythrocyte sedimentation rate at 58 mm/hour. Cervical enhanced computer tomography revealed multiple circular well-defined soft tissue masses in the bilateral carotid sheath spaces, with the largest one (28.2 mm × 17.8 mm in size) located under the jaw (Fig. 1A). A computer tomography scan of the chest showed no abnormal findings. Abdominal color ultrasound revealed no obvious abnormalities in the liver, gallbladder, pancreas, spleen, or kidneys. The enlarged cervical lymph nodes were suspected to be caused by lymph node tuberculosis. A cervical lymph node biopsy was performed. Bloody pus was extracted from the lymph node. Cytological evaluation showed caseous necrosis with infiltration of neutrophils and lymphocytes, and most importantly, mycobacteria through staining for acid fast bacilli (Fig. 1B). In order to further screen for any digestive tract tuberculosis, gastroscopy, and colonoscopy were performed. Colonoscopy did not reveal any abnormalities. However, gastroscopy showed a 0.5 cm × 0.5 cm well-defined erosion on the large curvature of the gastric body (Fig. 2A). Repeated deep excavation biopsy was performed and the tissue samples were stained with hematoxylin and eosin. Histopathological examination revealed chronic granulomatous inflammation (Fig. 2B and 2D). Mycobacteria were found in 6 consecutive tissue sections through staining for acid fast bacilli (Fig. 2C and E). Therefore, the patient was diagnosed as having cervical lymph node tuberculosis and gastric tuberculosis. She received an anti-tuberculosis therapy consisting of oral isoniazid (5 mg/kg once daily), rifampicin (10 mg/kg once daily), ethambutol (15 mg/kg once daily), and pyrazinamide (25 mg/kg once daily) in the initial 2 months, followed by isoniazide and rifampicin at the same dosages for 4 additional months. The patient's liver and kidney functions were closely monitored one week after treatment and then monthly. No remarkable side effects were observed. The enlarged cervical lymph nodes shrank in size and the pain was relieved. The treatment appeared effective.

## 3 Discussion and conclusion

Gastric tuberculosis is rare, which occurs mostly secondary to lung tuberculosis, intestinal tuberculosis, urinary tuberculosis, bone tuberculosis, or pelvic tuberculosis.^[2]^ It often occurs in people with underdeveloped economy and poor living conditions. The disease is more common in young and middle-aged people, especially women.^[1,2]^ The pathogenesis of gastric tuberculosis is not very clear. Several potential mechanisms have been proposed:

1. It develops secondary to any primary tuberculosis loci, such as the lungs and kidneys from which mycobacteria reach to and colonize in the stomach through blood and/or lymphatic circulations.
2. It occurs when mycobacteria colonize the stomach through oropharyngeal route in a patient with any mucosal barrier damage after gastric mucosa injury and reduced gastric secretion, thus the germicidal self-purification effect of the gastric juice is weakened; or, the patient has any disorders of gastric motility and decelerated evacuation, leading to prolonged time of mycobacteria staying in the stomach and forming an infection locus.
3. A tuberculosis locus in an adjacent organ spreads to and infiltrate through the gastric wall, thus forming a gastric tuberculosis locus.^[7,8]^

In this case, the patient had cervical lymph node tuberculosis, without any tuberculosis in the lungs or adjacent organs. Therefore, we speculate that her gastric tuberculosis was likely due to blood and/or lymphatic circulation of mycobacteria to the stomach. The clinical symptoms of gastric tuberculosis are non-specific. Small tuberculosis lesions in the stomach are often asymptomatic. Large lesions may show early symptoms similar to chronic gastritis, gastric ulcer, and gastric cancer, such as abdominal pain and abdominal distension. The patient may vomit if complicated with pyloric obstruction, while a few patients may have upper gastrointestinal bleeding. Gastric tuberculosis shows in a variety of forms that are non-specific under gastroscopy. Literature reports describe as irregular erosions, ulcers, or submucosal bulge lesions.^[9–12]^ In many cases, it is often misdiagnosed as gastric malignant tumors.^[4,6,9]^ Severe gastric tuberculosis can lead to gastric sinus deformation^[5]^ and pyloric obstruction.^[12]^ The antrum and prepyloric regions (especially the posterior wall) are the most common sites of gastric tuberculosis, which is presumably related to the fact that the gastric sinus area has mucosal pyloric glands in which mycobacteria tend to colonize.^[13–14]^ This case presented no clinical symptoms of the gastrointestinal tract and was found by routine gastroscopy screening. What unusual is that the small lesion may often be missed if not carefully examined. The diagnosis was made possible only after microscopic examination of the biopsied tissue with staining for acid fast bacilli. To the best of our knowledge, gastric tuberculosis of such a small lesion has not been reported in the literature.^[14]^ In conclusion, this case suggests that gastroscopy should be performed to look for gastric tuberculosis if the patient presents primary tuberculosis in other organs/tissues such as cervical lymph nodes. If any small erosion is found, a biopsy is justified for checking the possibility of gastric tuberculosis.

## Author contributions

**Data curation:** Wenguang Zhang, Fusheng Song, Zhimei Zhang, Jun Yang, Linlin Zhao.

**Formal analysis:** Zhimei Zhang, Jun Yang, Linlin Zhao.

**Project administration:** Linlin Zhao.

**Writing – original draft:** Wenguang Zhang, Linlin Zhao.

## References

[1] Chaudhary P Khan AQ Lal R Bhadana U. Gastric tuberculosis. Indian J Tuberc 2019;66:411–7. 31439189 10.1016/j.ijtb.2018.10.004

[2] Seetlani NK Imran K Hafeez QUA. Gastric tuberculosis. J Coll Physicians Surg Pak 2019;29:S20–2. 31142410 10.29271/jcpsp.2019.06.S20

[3] Zhu R Zhou Y Wang HDi L Zhao K Tuo B Wu H. Gastric tuberculosis mimicking submucosal tumor a case series. BMC Gastroenterol 2020;20:23. 32000710 10.1186/s12876-020-1175-x PMC6990527

[4] Lv M Tang K Meng Y Tian C Wang M. Primary isolated asymptomatic gastric tuberculosis of the cardia mimicking gastric stromal tumor: a rare case report and literature review. BMC Gastroenterol 2020;20:108. 32293275 10.1186/s12876-020-01242-x PMC7158059

[5] Arabi NA Musaad AM Ahmed EE Ibnouf MM Abdelaziz MS. Primary gastric tuberculosis presenting as gastric outlet obstruction: a case report and review of the literature. J Med Case Rep 2015;9:265. 26577440 10.1186/s13256-015-0748-8 PMC4650840

[6] Memon W Siddiqui S. Isolated gastric tuberculosis mimicking malignancy. J Ayub Med Coll Abbottabad 2016;28:814–5. 28586582

[7] Mukhopadhyay M Rahaman QM Mallick NR Khan D Roy S Biswas N. Isolated gastric tuberculosis: a case report and review of literature. Indian J Surg 2010;72:412–3. 21966145 10.1007/s12262-010-0133-1 PMC3077138

[8] Amarapurkar DN Patel ND Amarapurkar AD. Primary gastric tuberculosis--report of 5 cases. BMC Gastroenterol 2003;3:06. 10.1186/1471-230X-3-6 PMC155648 12703983

[9] Eray IC Rencuzogullari A Yalav O. Primary gastric tuberculosis mimicking gastric cancer. Ulusal Cerrahi Derg 2015;31:177–9. 10.5152/UCD.2014.2667 PMC4605117 26504425

[10] Kim SH Park JH Kang KH. Gastric tuberculosis presenting as a submucosal tumor. Gastrointest Endosc 2005;61:319–22. 15729256 10.1016/s0016-5107(04)02637-9

[11] Gupta V Goel MM Noushif M Rai P Gupta P Chandra A. Primary gastric tuberculosis mimicking gastrointestinal stromal tumor. Am J Gastroenterol 2012;107:1269–70. 22859010 10.1038/ajg.2012.120

[12] Bandyopadhyay SK Bandyopadhyay R Chatterjee U. Isolated gastric tuberculosis presenting as haematemesis. J Postgrad Med 2002;48:72–3. 12082338

[13] Lin OS Wu SS Yeh KT Soon MS. Isolated gastric tuberculosis of the cardia. J Gastroenterol Hepatol 1999;14:258–61. 10197496 10.1046/j.1440-1746.1999.01844.x

[14] Geo SK Harikumar R Varghese T Rajan P Aravindan KP. Isolated tuberculosis of gastric cardia presenting as perforation peritonitis. Indian J Gastroenterol 2005;24:227–8. 16361778

